# The Australian Roadkill Reporting Project—Applying Integrated Professional Research and Citizen Science to Monitor and Mitigate Roadkill in Australia

**DOI:** 10.3390/ani10071112

**Published:** 2020-06-29

**Authors:** Bruce Englefield, Melissa Starling, Bethany Wilson, Caidyrn Roder, Paul McGreevy

**Affiliations:** Sydney School of Veterinary Science, University of Sydney, Sydney, NSW 2006, Australia; melissa.starling@sydney.edu.au (M.S.); bethany.wilson@sydney.edu.au (B.W.); caidyrnr@gmail.com (C.R.); paul.mcgreevy@sydney.edu.au (P.M.)

**Keywords:** roving reports, mobile application, ecological studies

## Abstract

**Simple Summary:**

Australia has no database of national roadkill. The current research project fills that knowledge gap by developing a roadkill reporting application that enables professional and citizen scientists to record photographs of roadkill with location, time and date. This embodies the concept of ‘One Welfare’ as it affects humans, animals and the environment. Uploaded to a website, these data can identify roadkill hotspots, tabulate species of animals killed and potentially be used for ecological studies of roadkill numbers, species distribution, population trends, animal behaviour and disease. Initial results indicate that mammal roadkill mostly occurs at night and that of birds and reptiles during daytime. Mammals make up three-quarters of the roadkill recorded and this includes endangered species. Two examples of roadkill hotspots are shown in Queensland and Tasmania. These will enable further research to suggest how roadkill mitigation measures may be optimally employed.

**Abstract:**

Australia has no national roadkill monitoring scheme. To address this gap in knowledge, a roadkill reporting application (app) was developed to allow members of the public to join professional researchers in gathering Australian data. The app is used to photograph roadkill and simultaneously records the GPS location, time and date. These data are uploaded immediately to a website for data management. To illustrate the capacity to facilitate cost-effective mitigation measures the article focuses on two roadkill hotspots—in Queensland and Tasmania. In total, 1609 reports were gathered in the first three months of the project. They include data on mammals (*n* = 1203, 75%), birds (*n* = 125, 7.8%), reptiles (*n* = 79, 4.9%), amphibians (*n* = 4, 0.025%), unidentified (*n* = 189, 11.8%) and unserviceable ones (*n* = 9). A significant finding is variance in the distribution of mammals and birds at different times of day. These findings reflect diurnal variation in the activity levels of different species and underline the need for data on a targeted species to be collected at appropriate times of day. By continuing to facilitate roadkill monitoring, it is anticipated that the data generated by the app will directly increase knowledge of roadkill numbers and hotspots. Indirectly, it will provide value-added information on animal behaviour, disease and population dynamics as well as for species distribution mapping.

## 1. Introduction

Roadkill is a term that encompasses all mammals, birds, reptiles and amphibians that are killed by vehicles on roads. Although Australia lacks national data on roadkill, there have been state-based studies, notably in New South Wales [1,2,3,4,5], Tasmania [6], Victoria [7] and South Australia [8]. Dating from 1972 through to 2008, these were conducted by professional researchers. However, emergent technologies, such as mobile phones, mean that the collection of reliable roadkill data has become possible for volunteer citizens. Such volunteers have become known as citizen scientists, a term coined in 1995 by Rick Bonney in the USA [9] and Alan Irwin in the UK [10]. These technologies bring with them many advantages, particularly when the area of environment to be studied is as geographically broad as Australia. Citizen scientists can gather data on a scale beyond the reach of most research budgets. 

It is acknowledged that there are problems and pitfalls with the recruitment, motivation and retention of volunteers, ensuring safe working practices and the quality of the data produced [11,12], but these challenges can be overcome [13]. An increase in citizen science projects from 100 in 2011 to 700 in 2018 is reported by SciStarter [14] and this is reflected in the increasing number of peer-reviewed papers published annually that use or include data from citizen scientists [15,16].

Examples of projects that use such data include the *doglogbook*, developed by the dogmanship team at the Sydney School of Veterinary Science, University of Sydney, Australia [17]. This aims to support evidence-based assessments of dog quality of life and enhance dog welfare in clinical practice. Outside the realm of domesticated animals, other examples are Birdata, run by Birdlife Australia [18], and eBird, run by Cornell University [19]—both of which collect information on birdlife supplied by citizen scientists. 

Records made whenever and wherever an observer observes a target, such as roadkill, are called roving records [13]. National roadkill monitoring projects using roving reports are in place across the globe, but some, being fairly undersubscribed, are limited in their scope. Examples include ‘Project Splatter’ [20] and ‘Mammals on Road’ [21]—both of which are in the UK—‘Roadkills’ in India [22], ‘Roadkill Observation Network’ in Japan [23], ‘Animals under Wheels’ in Belgium [24], Srazenazver.cz in the Czech Republic [25] and ‘Projekt Roadkill’ in Austria [26]. 

The challenge of obtaining reliable data gathered by volunteers with only basic training can be overcome by emergent smartphones and tablets and the development of applications (apps) that can be downloaded to them. Many smartphones have in-built GPS receivers and can access environmental information through open-source databases such as Google Maps that offer satellite imagery, aerial photography, street maps, 360° interactive panoramic views of streets, real-time traffic conditions, and route planning for traveling by foot, car, bicycle, air or public transportation. The technological advantage of obtaining accurate data through apps is that no knowledge of computer technology is required to use them and they are mobile. It has been shown that these apps can be used to improve data collection by both professional researchers [27] and citizen scientists [28,29]. 

Potential shortfalls in the collection of accurate ecological data include the inaccurate recording of time and location, recording duplication of data, incorrect identification of subjects and even loss of data records. For roadkill studies specifically, numerous constraints on the collection of data reflect temporal, spatial and taxonomic challenges that can cause gaps in knowledge. If citizen scientists are to be used in a roadkill monitoring research programme, the method of data collection must be as fail proof as possible and produce data that are incontrovertible. 

In essence, this means that a citizen scientist engaged in recording roadkill needs no skills other than to be able to spot roadkill and then use the app as directed. A photograph of the roadkill that is digitally stamped with GPS location, time and date and then uploaded to a website would meet these criteria. In contrast to relying on paper hard copy, the use of a tablet or smartphone has the advantage over paper hard copy that the observations are recorded swiftly and accurately, and are automatically uploaded to a website. If no mobile coverage is available, the app can record the GPS via satellite and then upload recorded roadkill data to a website once mobile coverage is obtained. The user does not need to separately record a GPS from a tracker, the time and date from a secondary source and then write the results into a field notebook—no easy task in wind, rain or snow. These notes have then to be copied to a website, a process that may allow errors to creep in. 

The current project followed the exemplar of mobile application for recording roadkill reported in other countries including the UK [20], USA [30], Belgium and Austria [31] Columbia [32] and Tanzania [33]. The functional specifications were for the current Australian Roadkill Reporting Project (ARRP), using the Roadkill Reporter App (RRApp), to track Australian roadkill by means of a smartphone or tablet by a researcher or citizen scientist. 

The long-term aim of the project was to provide an estimate of the scale of the national roadkill problem and produce a baseline of annual roadkill data with which future roadkill data could be compared. 

## 2. Materials and Methods 

### 2.1. A Review of Roadkill Reporting Applications

A review of the functionality of existing roadkill reporting apps, undertaken in early 2017, demonstrated that all were inadequate for the purpose of this study or suffered from prohibitive leasing costs. A new app, named the Roadkill Reporter App (RRApp), was, therefore, developed locally.

### 2.2. Features and Functionality of the RRApp

It was important that the RRApp fulfilled the following criteria:

- Must be easy to use, requiring no technical knowledge to operate;
- Freely available;
- Portable and accessible;
- Functional on Apple iOS and Android^TM^ mobile operating system;
- Data on the app must be able to be uploaded to a secure website via a web facility to collate, store and allow access through a website interface to data submitted through use of the mobile application;
- Data collected must include a photograph and GPS, time and date;
- Users must have the ability to review trends by accessing the website;
- Users must be able to make notes to accompany a roadkill photograph;
- Users must remain anonymous.

The website for monitoring the app was required to be hosted in Australia if possible.

### 2.3. Usage and Testing of the RRApp

The app allows the use of personal mobile devices to take a photograph of roadkill and submit it to a cloud-based database of sightings along with the time of day and GPS location.

Once developed, the app was subjected to testing by a mixed group of volunteers (*n* = 14) of different gender, age and familiarity with the use of information technology. Feedback from this testing resulted in refinements being introduced to ensure the RRApp was user-friendly for citizen scientists of different abilities. The participants were required to take a photograph of the roadkill, categorise it as mammal, bird, other or splat (unidentifiable roadkill) and then submit the report. The app ensures that the GPS coordinates and time of day are automatically submitted.

The RRApp was further tested in a pilot study during a research trial of a virtual fence system [34]. Duplicate data were recorded on 10 occasions over three consecutive days, using both the RRApp and independent GPS tracking and a digitally stamped time and dated photograph. A concordance correlation coefficient and standard deviation were calculated for the GPS data and the time and date separately compared to confirm the accuracy of the RRApp was within required spatial limits.

### 2.4. Ethics Exemption and Launch of the RRApp

The project was exempted from ethics review and a Roadkill Reporter Exemption certificate was issued by the Research Integrity and Ethics Administration, Human Research Ethics Committee of the University of Sydney (Appendix A). 

The RRApp was launched in September 2019 through national and local television, radio, newspapers, social media and wildlife carer networks. The data are used to produce a map of recorded roadkill that can be accessed at www.roadkillreporter.com.au/reports. The map is updated in real time and is open access, so that any individual can view their uploaded sightings as well as those reported by others. 

### 2.5. Analysis of Roadkill Data

The normal choice for count data is a Poisson model but, in this case, the data were overdispersed (i.e., the variance was greater than the mean), so a quasi-Poisson model was used. Modelling was performed in the R stats package. The results were wrapped in an ANOVA wrapper using the car package [35]. To determine what time of day roadkill was being recorded using the RRApp, the quadrant of day (QOD) coefficients were graphed in R with a 95% confidence interval using jtools [36]. The emmeans package [37] was used to calculate estimated marginal means and asymptotic confidence intervals, and attain pairwise *p*-tests. The level of significance was set at *p* ≤ 0.05

Locations were categorised into states and territories manually using the state borders from the Australian Bureau of Statistics [38]

The reporting time was recorded as Universal Time Coordinated (UTC). This is the time standard commonly used globally and recorded by the RRApp. This was converted to local time based on the state boundaries and daylight-saving protocols specific to each state.

The time of day was divided into 4 quadrants rather than either 24 h or as a continuous variable due to low numbers of data. The quadrants were ordered in six-hour blocks starting from midnight: 00:00–05:59 Quadrant 1 (Q1), 06:00–11:59 Quadrant 2 (Q2), 12:00–17:59 Quadrant 3 (Q3), and 18:00–23:59 Quadrant 4 (Q4).

Experts in herpetology (*n* = 4), ornithology (*n* = 4) and zoology (*n* = 5) examined reported photographs and used the location and respondents’ notes, together with their own expertise, to subjectively produce a percentage certainty between 1% and 100% confidence of the accuracy of their species identification of each image. Only where two or more experts had a confidence of greater than 90% was identification ratified; otherwise, the species was recorded as unknown. Sensitivity and specificity were analysed by comparing the user identification of a photograph as a mammal to the expert identifying a photograph as a mammal. The purpose of these identifications was to assess the accuracy of user identifications. Future users of data collected by this app may find such statistics useful for estimating sample sizes and efficiently allocating research resources such as availability of taxonomic experts.

Using the webmap of Australian roadkill produced from the citizen scientist observations of roadkill from the RRApp, it was possible to identify transects where there was a high density of roadkill. These transects were extracted and individually analysed. 

Two clusters of roadkill on transects in Queensland and Tasmania, where roadkill was apparently high, were selected for detailed examination using the webmap produced by the RRApp website.

## 3. Results

### 3.1. Assessment of the Accuracy of the Roadkill GPS Using the RRApp

Ten GPS locations of roadkill were recorded on both the SMGPS app and the RRApp and compared for concordance (Appendix B). Both the standard deviation (1.739) and product moment correlation coefficients (Easting 0.999999231 and Northing 0.999997278) demonstrate that the RRApp performed well within the accuracy required in the specifications.

### 3.2. Overview of the RRApp from 1 September to 30 November 2019, Installation of the RRApp and Subsequent Reports

In total, 2318 people installed the RRApp (*n* = 1445 on iOS and *n* = 873 on Android). There were 1459 reports made, with 322 users making at least one report. Approximately 14% of people who installed the app made at least one report. There was an average of 4.5 reports per user and 39% of users made just one report. Ten per cent of the users were responsible for 50% of reports. 

The purpose of this analysis was to uncover diurnal patterns in the reports. This was useful to explore the time of day users are using the app and also when roadkill deaths are reported. The importance of this was to understand whether roadkill, particularly avian and reptilian, that occurred during the daytime was being recorded before being removed by anthropogenic means or by animal scavengers.

**Figure 1:**
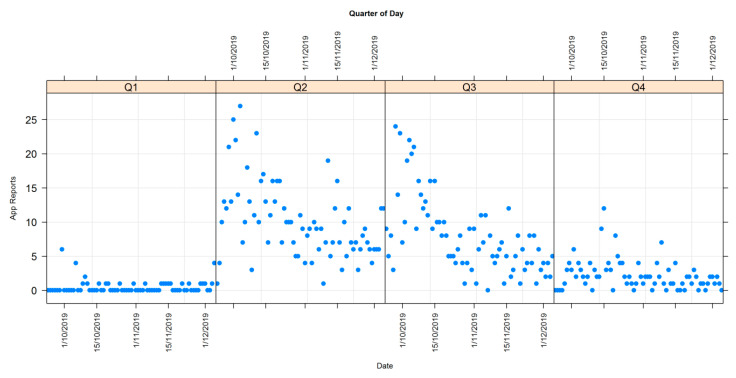
Distribution of roadkill reports by time of day, divided into four quadrants of day.

Reports from 24/9/2019 to 5/12/2019 are more common in the later morning (Q2, 6:00 a.m. to midday) and in the afternoon (Q3, midday to 6:00 p.m.), with a moderate number of reports in the evening (Q4, 6:00 p.m. to midnight) and the fewest in the early morning (Q1, midnight to 6:00 a.m.) (Figure 1).

Both the quadrant of day (QOD) (LR χ^2^ = 482.38; *p* < 0.001) and date (LR χ^2^ = 66.8; *p* < 0.001) were significantly associated with the number of reports made. The number of reports declined towards the end of the study period.

Q1 (early morning before 6:00 a.m.) was associated with the lowest number of reports, significantly lower than Q2 (6:00 a.m. to midday) (r = −3.046, z = 12.013; *p* < 0.001), Q3 (midday to 6:00 p.m.) (r = −2.804, z = 10.992; *p* < 0.001), and Q4 (6:00 p.m. to midnight (r = −1.520, z = 5.557; *p* < 0.001). Q4 was also significantly lower than Q2 (r = −1.526, z = 11.937; *p* < 0.001) and Q3 (r = −1.284, z = 9.810; *p* < 0.001). Of the two daylight quadrants, Q2 had significantly more reports (r = 0.242. z = 2.967, *p* = 0.016) than Q3. 

**Figure 2:**
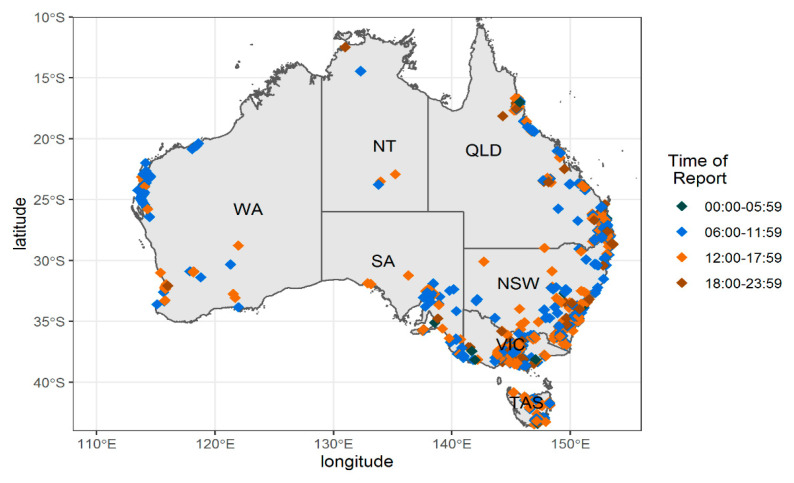
Location of roadkill reports by the quadrant of day (QOD), superimposed on a map of Australia. WA—Western Australia, NT—Northern Territory, SA—South Australia, QLD-Queensland, NSW—New South Wales, VIC—Victoria, and TAS—Tasmania.

The location of reports by the local QOD suggests that the RRApp is widely used but that the Northern Territory is under represented (Figure 2). This is demonstrated on a state by state basis (Table 1).

### 3.3. Reported Class of Roadkill during Study Period

Roadkill were classified by RRApp users into mammal (class Mammalia), bird (class Aves), other (any other class) or splat (unidentifiable remains). Data for 1509 reports taken between 24 September 2019 and 5 December 2019 UTC are shown in Table 2.

The most commonly reported class of roadkill were mammals, comprising 81.3% (1227/1509) of reports. The next most common were birds, accounting for 9.0% (135/1509) of reports. Other identified remains accounted for 8.0% (120/1509) of reports and unidentified reports as the remaining 1.8% (27/1509). A Fisher’s exact test revealed that the taxonomic class of the roadkill report was associated with the time of day of the report (*p* < 0.001). Post-hoc tests showed that the proportion of roadkill by class differed across all pairwise comparisons, including between the two “daylight” quadrants (Q2 and Q3; z = 2.967, *p* = 0.016) and between the two “night’ quadrants (Q1 and Q4; z = 5.557, *p* < 0.001).

### 3.4. Accuracy of Observers Recording Mammal, Bird, Other, and Splat

Experts were able to characterise all reports recorded as splat (*n* = 25) as either mammalian, bird or other (Table 3). 

The roadkill (*n* = 175) identified as not otherwise classifiable represent a combination of results from experts who were able to identify the class but not the species (therefore, unable to classify whether it was native or exotic, macropod or not macropod, nocturnal or diurnal, etc.)

### 3.5. Accuracy For Mammals, Birds and Other

The sensitivity with which a user ‘mammal’ report predicted a mammalian roadkill photo was 0.980 and the specificity with which a user report of ‘mammal’ predicted a mammalian roadkill photo was 0.915. The sensitivity with which a user ‘bird’ report predicted an avian roadkill photo was 0.986 and the specificity with which a user report of ‘bird’ predicted an avian roadkill photo was 0.995. Finally, the sensitivity with which a user’s ‘other’ report predicted an amphibian or reptilian roadkill photo was 0.944 and the specificity with which a user’s report of ‘other’ predicted an amphibian or reptilian roadkill photo was 0.981.

### 3.6. Animal Species Represented in Roadkill

Images from 1609 reports between 28 September 2019 and 31 December 2019 were examined to identify the species of roadkill reported. In total, 13 reports were immediately discarded as duplicates (*n* = 6) or miscellaneous (*n* = 7). The main species represented in the roadkill photographs were kangaroos (*n* = 415, 26.74%), wallabies (*n* = 360, 23.19%) and wombats (*n* = 181, 11.66%), which collectively represented 61.59% of the total roadkill (Table 4). 

Only the main taxa in each category are shown in Table 4. A full analysis of the roadkill represented in the report photographs is available in the Appendix (Table C1). 

### 3.7. Roadkill Hotspots

**Figure 3:**
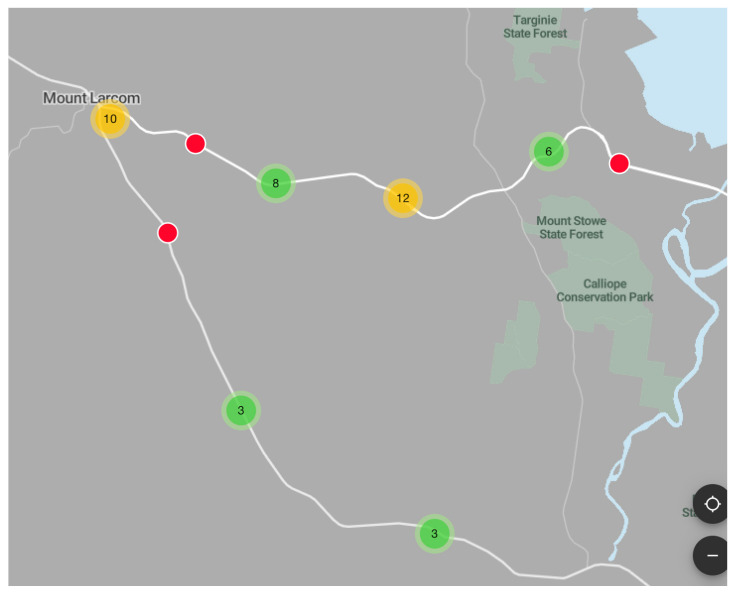
Roadkill hotspot on 20 km of highway between Mount Larcom and east towards Mount Stowe State Forest, Queensland.

Two transects of highways where roadkill was apparently high were selected for detailed examination: one in Queensland and the other in Tasmania. On the Queensland transect, the roadkill total (*n* = 38) on 600 km of road observed (20 km × approximately 30 days of monitoring by citizen scientists) was calculated to be a rate of 0.063 roadkill·km^−1^·day^−1^ (Figure 3).

The red circles represent one roadkill, the green dots represent between three and nine roadkill and the yellow dots represent upwards of ten roadkill. 

**Figure 4:**
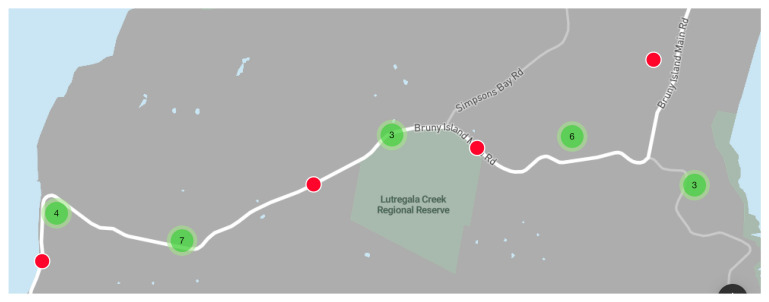
Roadkill hotspot on 6 km of highway between Alona on the west side and Adventure Bay on the east side of Bruny Island Tasmania.

On the Tasmanian transect, the roadkill total (*n* = 25) on approximately 360 km of road observed (6 km × approximately 60 days of monitoring by citizen scientists) was calculated to be at a rate of 0.069 roadkill·km^−1^·day^−1^ (Figure 4).

The red dots represent one roadkill and the green dots between two and nine roadkill. 

## 4. Discussion

There are several ecological and human reasons for gathering data on Australian roadkill. ‘One Welfare’ is an emerging term. The concept refers to animal welfare, human welfare and environmental sustainability. It serves to highlight the interconnections between animal welfare, human wellbeing and the environment, and promotes direct and indirect links [39,40]. Roadkill can be placed in the concept of ‘One Welfare,’ as it affects humans, animals and the environment. The current project demonstrates the advantages and benefits of comprehensively, effectively and systematically monitoring Australian roadkill as a ‘One Welfare’ concept. The data obtained can be used to identify roadkill hotspots, raise the profile of roadkill in the mind of the public, identify the species killed and determine their distribution. This information can be entered into the database for future evaluation. 

Engaging citizen scientists has highlighted the problems that roadkill presents in the mind of the general public through the publicity it has attracted. Evidence of this can be seen in the sudden increase in installations during September and reports at the beginning of November 2019 correlating with media exposure. As the project progresses, this public awareness is likely to increase. Community groups will be able to use the data from the RRApp to enhance research and general funding for mitigation measures and their implementation. The project has also shown how the time of monitoring by citizen scientists is dependent on the quadrant of day (QOD), i.e., mainly between 06:00 and 18:00. This would indicate that it might be advantageous to target members of those demographic groups, such as wildlife carers, so-called grey nomads (Australian retirees who travel within their own country for an extended time, usually in a caravan or motor home), local councils and environment networks who are travelling or working during this time (Q2 and Q3). The data gathered can also be used in ecological studies. 

The data shown in Figure 2 are based on state boundaries, and this process draws in some minor idiosyncrasies. Specifically, it does not account for the small area in the southeast of Western Australia that borders South Australia that is on Australian Central Western Standard Time or the fact that Broken Hill (New South Wales) is on Adelaide time (Australian Central Standard Time).

Five areas of how monitoring roadkill can be used are explored in depth by Shilling and Perkins [41]. These areas are: monitoring wildlife roadkill numbers; species distributions; population trends and impacts; animal behaviour (specifically movement); and contaminants and disease. In the context of the current project, it has been shown how these five areas are pertinent to Australian ecology.

The current mapping outputs (Figure 3 and Figure 4) confirm that by citizen scientists monitoring wildlife roadkill numbers, transects where roadkill seems high can be identified as hotspots. Although the project is in its early stages, it has been possible to use some of the data to indicate important species distributions. One example is the reported distribution of cane toads *(Rhinella marina*, *n* = 4)—three reports of which were from Queensland and one of which was apparently from northern New South Wales—this latter report is at the limit of the known range of this introduced pest species. Future roadkill sightings could alert authorities to further spread of this species which has been predicted to stretch to the Australian west coast [42]. Should increasing global temperatures make habitat available to cane toads even further south than is currently predicted, it is important to use every method available, such as the simple and inexpensive RRApp, to detect this possible movement. 

The current data indicate that over time, collated roadkill reports can highlight relative population trends. One example is that of the Tasmanian devil (*Sarcophilus harrisii*). Only a single roadkill was reported by RRApp users (Appendix C). This low number could reflect the ongoing impact of Devil Facial Tumour Disease (DFTD). This is an aggressive non-viral clonally transmissible cancer which affects Tasmanian devils which is associated with high mortality [43]. As DFTD has reduced the Tasmanian devil population, there has been a drop in Tasmanian devil roadkill numbers [6,44,45]. A higher reported number of roadkill devils in longitudinal data might indicate a species recovery, as other authors have used roadkill as an indicator of population size or density. For example, Baker et al. used road traffic casualties (roadkill) to monitor population changes in red foxes *(Vulpes vulpes)* [46], Canova and Balestrieri to monitor mammal species [47] and Gehrt to monitor raccoon abundance [48]. Similar data could be used to monitor feral cat numbers in Australia. The discovery of foxes *(Vulpes vulpes)* in Tasmania was triggered by a roadkill sighting, as happened in the early stages of the successful fox eradication programme in the island state [49,50,51]. In the re-introduction programme of the eastern quoll *(Dasyurus viverrinus)* to the mainland of Australia, many quolls became roadkill [52]. With over 2000 roadkill reports in three months from the RRApp, there exists an initial baseline against which longitudinal trends can be analysed in the future. 

The current data hold some promise for revealing how species adapt their behaviour to the rapid environmental changes caused by bushfires, drought and flood [53,54]. Monitoring of roadkill can give an insight into behavioural changes, such as animal movement. In a country as large as Australia, RRApp data from citizens already on the move could provide a means to reveal behaviour changes that would otherwise be expensive and difficult to undertake. 

Photographs of roadkill can also be used to monitor the incidence of disease in affected animals. Sarcoptic mange in wombats [55] and DFTD in Tasmanian devils [56,57], for example, can both be successfully detected using roadkill photographs. Such photographs, combined with their date- and time-stamped GPS can be used as sentinels to monitor wildlife disease. Sarcoptic mange is the biggest killer of wombats, and is reported to be present in 90% of common wombat populations. However, from examination of the photographs of reported roadkill wombats (*n* = 181), it was not possible to identify a single case of mange. This suggests that infected animals may not present on roadsides to feed and expose themselves to vehicles and become roadkill.

A further benefit of data from RRApp is the ability for Australia to join the other 12 countries undertaking roadkill reporting schemes and to submit data to the Global Biodiversity Information Facility (GBIF) (https://www.gbif.org/), a database that currently (March 2020) holds approximately 1.5 billion records and 49,050 individual datasets [41]. Australia is a voting participant on the GBIF. Publishing data from the RRApp, into the *Atlas of Living Australia* [58] the national node, should result in those data being published globally through the GBIF. The benefits of doing so are explored by Edwards [59], and include allowing users to search for georeferenced specimen and observational data, check for common and scientific names for organisms (including synonyms), plot maps showing the known localities of specimens in the system, and retrieve lists of taxa by country. 

There are several limitations to using citizen scientists to gather roving reports and there is also concern regarding the rigor and usability of data collected by citizens who have not been formally trained in empirical science [60]. These merit detailed consideration. Recruitment and sustainability of volunteers can be a restraint on the quantity of data gathered. This is shown in this project by the reduction in reports logged per day over time, which infers a reduction in participants from the launch in September to November 2019. Media coverage was successful in driving initial uptake in Oct (*n* = 812), but usage dropped off considerably in November (*n* = 443). The number of reports seemed to be stabilizing, with approximately *n* = 380 in December 2019 and *n* = 368 in January 2020. 

There was a drop off between the 2318 people who installed the RRApp between September 2019 and December 2019 and the 322 who produced a report. These data showed that approximately 10% of the users were responsible for approximately 50% of the reports. This indicates that there is a subset of users who are highly engaged with the app. In addition, potential reporters have to possess and operate a smartphone, iPad, tablet or similar device and to be able to download the RRApp. The population of Australia over the age of 14 years in 2020 is estimated at 20,500,000 [61]—of whom, 18,880,000 [62] have smartphones. However, despite 91% of the Australian population over the age of 14 owning smartphones, rates of engagement with the app remain an issue.

Another constraint on gathering data is that the time of day that the recording takes place is at the discretion of the user. Citizen scientists do not actively report the absence of roadkill and they may travel along a transect and take no photograph. This may skew results, as it is not possible to know the frequency with which any given transect is monitored. Thus, only an approximate calculation can be made of the roadkill rate per kilometre per week. The inability to calculate effort put in by each observer remains problematic for most citizen science projects, although the literature does suggest a number of ways that this can be addressed [63,64,65]. The data in Figure 1 indicate that most reports were made between 06:00 and 18:00 (Q2 and Q3). Mammal reports appear to account for a higher percentage of midnight to midday reports whereas bird reports appear disproportionately high between midday and midnight. “Other” reports (reptilian) appear most common in the afternoon and evening. The significance of these findings is that they align with the reality that most of Australian mammals killed on the roads are crepuscular or nocturnal feeders [2,6]. This means that the most advantageous time to record mammalian roadkill is after dawn and before carcasses are dispersed by scavengers or anthropogenic means. In contrast, avian and reptilian roadkill tend to be active day feeders and reptiles that use the warming tarmac of roads to thermo-regulate before hunting, so most are likely to be killed between dawn and dusk. Thus, the most advantageous time to monitor avian and reptilian roadkill is before dusk. 

Another consideration is the in situ persistence of roadkill. The lower the mass of individual roadkill, the shorter the time it persists in the environment [66]. Therefore, avian and reptilian roadkill need to be recorded as soon as possible after death, i.e., evening time. In controlled studies of roadkill among targeted species, the observation and recording time would be chosen to suit the species being monitored; a provision unlikely to be available for citizen science projects in which volunteers are likely to monitor at a time suited to themselves. In the current project, most reports were taken in Q2 and Q3. Although the highest number of avian and reptilian roadkill reports occurred in Q4, the evening (Table 1), the overall percentage of avian and reptilian roadkill relative to mammalian roadkill was 14.75% to 85.25%, respectively. 

A further limitation of citizen science could be the possible need to train the citizen scientists. However, the RRApp requires virtually no training to use it, so it is extremely efficient in use of respondents’ time. However, it does require experts to donate their time for identification of animals but many organisations such as iNaturalist, the Australian and State museums as well as the National Parks and Wildlife service offer complimentary species identification services. These services could be utilised to make the ARRP sustainable.

It is also worth acknowledging that the current data may include some observer bias. People are more likely to see larger roadkill than smaller, and may be more interested in mammals than reptiles/birds. The local speed limit of the road might also have an impact on ability to observe, pull over and record. The use of the RRApp whilst driving a vehicle would render the user liable to prosecution. So, a solo driver is unable to use the RRApp unless they are able to stop the vehicle in a safe place. This adds to one’s journey time, so may discourage the driver from recording data. Clearly, if the vehicle carries on moving, a passenger must operate the RRApp. This presents a further limitation because the resultant photographs would be taken from some distance and could be blurred, which in turn makes identification of roadkill at a species level unreliable. Similarly, depending upon the speed of a vehicle, smaller animals may go unseen and unrecorded.

There are important differences in potential reporters’ opportunity to pull over and record data. Some roads have no safe shoulder on which to pull over. It may be that roads with high speed limits [67], that are winding or uneven, are more likely to have high roadkill but are also more difficult for citizen scientists to pull over in a safe and timely fashion after spotting roadkill, so compromising the representativeness of data collection. However, data that are collected can be used to produce a general map that may help identify a possible roadkill hotspot transect. The data recorded are the minimum roadkill that occurred so produce a conservative estimate of roadkill. Nevertheless, some hotspots will inevitably be missed through lack of data. For the two hotspots analysed here, from Queensland and Tasmania (Figure 3 and Figure 4), even the conservative results show a significantly higher rate of roadkill·km^−1^·day^−1^, at 0.063 and 0.069, respectively, than the average roadkill recorded in other science-based projects such as 0.031 [68], 0.035 [1] and 0.042 [6]. However, these other studies revealed marked temporal and spatially differences in topography and road conditions and were not focussed on identifying hotspots. Thus, it is reasonable to conclude that the two transects discussed in Queensland and Tasmania can reliably be defined as roadkill hotspots. 

Another limitation is the possibility of double counts of roadkill but, by using an algorithm to identify proximate GPS locations and viewing individual photographs contained in the RRApp recorded on the website, this problem was mitigated during analysis and duplicates (*n* = 6) were discarded. However, there is a positive aspect to duplicates in that they could be used to estimate persistence of roadkill and duplicates may present an image that improves the potential for animal identification. 

As indicated above, there are several possible avenues for future research. Once analysis of the RRApp reports has identified a cluster on a transect as a roadkill hotspot, it needs to be followed up by detailed research. Ideally, this should be supervised by professional researchers so that observer effort is standardised. Other areas that could be investigated, but would also require professional supervision on the use of the RRApp, include estimated latency between the roadkill event and the report, the relationships among day of the week, vehicle density and speed and roadkill [6,35,67,69]; the effectiveness of existing mitigation strategies (such as roadside fencing, wildlife bridges/crossings and culverts) [70,71,72]; and the role of normal species behaviour [73,74] (such as daily foraging/hunting, mating/nesting season and migration). 

Several possible refinements to the RRApp merit consideration. When recruiting users of the RRApp, the current project targeted the general population through television, newsprint and social media. The problem of volunteer recruitment and retention of citizen scientists has been addressed in several studies [75,76,77,78,79]. Bil et al. [80] suggest that volunteers need to be motivated by the organisers to participate on a long-term basis and be provided with regular feedback on how their data are being used to produce new scientific knowledge. They also suggest that the target group for recruitment are road users who have an interest in animal welfare, nature conservation or road safety and have access to the internet and a smartphone. This group could include environmentalists, hunters and shooters, local council road maintenance crews, police, drivers, commuters, grey nomads, wildlife carer groups, students and walkers. To reach the different groups that Bil et al. [80] suggested, it may prove necessary to recruit a broader cohort of citizen scientists. This could be achieved through editorial and talking point items in magazines as well as television and radio items and newspaper feature magazines. In addition, a comprehensive dedicated website that disseminates information gained may retain recruits and engage them, perhaps with feedback on reports received. There is also a need to engage a principal organisational entity such as a university, museum, or non-government organisation to take over the running of the ARRP. The danger of leaving it to one private individual to oversee could result in the rapid demise of the project that underpins the RRApp. One strategy to be explored would be to secure funding to build a team of professionals similar to that of the ebird project (*n* = 25) [19].

## 5. Conclusions

The ARRP collects substantial roadkill data that contribute to knowledge across a diversity of research needs. The current RRApp data report on roadkill in mammals (*n* = 1203, 75%), birds (*n* = 125, 7.8%) reptiles (*n* = 79, 4.9%) amphibians (*n* = 4, 0.025%) and unidentified (*n* = 189, 11.8%). A significant finding is variance in the distribution of mammals and birds at different times of day (*p* < 0.001). This paper also reveals how clusters of roadkill are identified in Queensland and Tasmania and how the presence of invasive species such as the cane toad, red fox and feral cat can be demonstrated through roadkill sightings. To reveal longitudinal trends, recruitment of citizen scientists and their retention over several years are required. Together, these can provide valuable data that could be used to instigate cost-effective roadkill mitigation measures, track animal behaviour and disease, monitor animal re-introduction programmes and the dispersal of feral species.

## Figures and Tables

**Table 1:** Total number of reports by state between 24/9/2019 and 5/12/2019 UTC.

STATE	Mammal	Bird	Other	Splat	TOTAL
NSW	295	28	39	6	368
NT	3	1	2	0	6
QLD	389	41	35	7	472
SA	63	10	7	1	81
TAS	147	8	18	7	180
VIC	245	35	4	3	287
WA	85	12	15	3	115
TOTAL	1227	135	120	27	1509

**Table 2:** Taxonomic class of 1509 roadkill reports from RRApp users according to the local quadrant of day of the report.

Time of Day	Mammal, *n* (%)	Bird, *n* (%)	Other, *n* (%)	Splat (Unidentifiable), *n* (%)
00:00–05:59 (Q1)	30 (85.71%)	3 (8.57%)	1 (2.86%)	1 (2.86%)
06:00–11:59 (Q2)	639 (86.82%)	50 (6.79%)	35 (4.76%)	12 (1.63%)
12:00–17:59 (Q3)	445 (76.99%)	58 (10.03%)	63 (10.90%)	12 (2.08%)
18:00–23:59 (Q4)	113 (70.63%)	24 (15.00%)	21 (13.13%)	2 (1.25%)
TOTAL	1227	135	120	27

**Table 3:** Experts’ identification of roadkill from photographs and how RRApp users identified those same roadkill.

Expert Identification	User Identification				
	Class	Mammal	Bird	Other	Splat
mammal—macropod	907	894	2	5	6
mammal—non-macropod	284	275	1	5	3
nocturnal bird	13	0	13	0	0
reptile	79	4	0	74	1
not otherwise classifiable	175	126	18	18	13
bird	110	0	108	1	1
exotic	25	14	2	8	1
amphibian	3	0	0	3	0

**Table 4:** Principal roadkill taxa between 28 September 2019 and 31 December 2019 by group, identified by experts using photographic RRApp data.

Group/Species		Number Recorded	Percentage of Total Roadkill
Mammals	Kangaroo (*Macropus*)	415	26.74
	Wallaby (*Macropus*)	360	23.19
	Wombat (*Vombatus ursinus*)	181	11.66
	Wallaroo (*Macropus robustus*)	77	4.96
	Brushtail possum (*Trichosurus vulpecula)*	48	3.10
	Koala (*Phascolarctos cinereus*)	17	1.09
	Ringtail possum *(Pseudocheirus peregrinus)*	9	0.58
Birds	Forest raven (*Corvus tasmanicus*)	16	1.03
	Brush turkey (*Alectura lathami*)	15	0.97
	Magpie *(Gymnorhina tibicen)*	14	0.90
Reptiles	Eastern brown snake (*Pseudonaja textilis*)	15	0.97
	Blue-tongue lizard (*Tiliqua*)	14	0.90
	Lace monitor lizard (*Varanus varius*)	11	0.71
	Red-bellied black snake (*Pseudechis porphyriacus*)	9	0.58
Exotics	Hare (*Lepus*)	6	0.39
	Cane toad (*Rhinella marina*)	4	0.26

## Appendix A. Human Research Ethics Exemption Certificate

Roadkill Reporter Exemption certificate

Research Integrity & Ethics Administration

Human Research Ethics Committee

Wednesday, 9 October 2019

Mr Bruce Englefield

Veterinary Science

The University of Sydney

Email: beng3578@uni.sydney.edu.au

Dear Bruce,

Project Title: Roadkill Reporter App

The NHMRC National Statement on Ethical Conduct in Human Research (“The National Statement”) outlines circumstances where research that carries only negligible risk may be exempted from ethical review.

The National Statement defines negligible risk as follows:

“The expression ‘negligible risk research’ describes research in which there is no foreseeable risk of harm or discomfort; and any foreseeable risk is no more than inconvenience.” (National Statement 2.1.7)

Further, the National Statement states that institutions may choose to exempt research from ethical review which meets the following criteria:

(a) “is negligible risk research (as defined in paragraph 2.1.7); and
(b) involves the use of existing collections of data or records that contain only non-identifiable data about human beings.” (*National Statement 5.1.22*).

Based on what you have described in communication with the Ethics Office, your project is negligible risk and will utilise a dataset from the Roadkill Reporter App that does not contain any identifiable information about human beings. Therefore, your project can be exempt from ethics review.

Should any future work not comply with any of the above, the project must be submitted for ethical review prior to commencing research. Please note that retrospective ethical approval of research cannot be given by the HREC.

Please contact the Ethics Office should you require further information or clarification.

Sincerely,

Karyn Ridgway

Human Ethics Officer

Research Integrity & Ethics Administration

Research Portfolio

Level 3, F23 Administration Building

The University of Sydney

NSW 2006 Australia

## Appendix B

**Table B1:** Standard deviation, Easting and Northing difference correlation co-efficient for comparisons (*n* = 10) of accuracy of RRApp taken by SMGPS app and the RRApp.

**Location**	**Share My** **GPS_SEasting**	**Share My** **GPS_SNorthing**	**RRApp** **_SEasting**	**RRApp** **_SNorthing**	**Distance between Locations in Metres**
1	520808.0227	−4757145.856	520806.3719	−4757146.984	1.999
2	520467.1664	−4757095.557	520465.2106	−4757097.795	2.972
3	516754.7728	−4758660.936	516756.1234	−4758658.762	2.559
4	516696.15	−4758687.444	516694.418	−4758688.85	2.231
5	517173.7803	−4758644.561	517175.3993	−4758639.456	5.355
6	517075.1475	−4758647.801	517075.6634	−4758646.892	1.045
7	518128.9989	−4758205.725	518130.5364	−4758204.019	2.297
8	520345.0792	−4757104.233	520350.832	−4757103.118	5.860
9	520483.7514	−4757074.752	520484.4861	−4757074.499	0.777
10	520777.6065	−4757136.758	520777.2204	−4757137.689	1.008
Easting Correlation Coef.: 0.999999231; Northing Correlation Coef.: 0.999997278; STD DEV: 1.739					
**Location**	**Easting—Easting** **Point 1**	**Northing—Northing** **Point 1**	**RRapp_Easting—Easting** **Point 1**	**RRappNorthing—Northing** **Point 1**	
1	0	0	−1.650797029	−1.127687	
2	−340.856288	50.29840563	−342.8121016	48.06106037	
3	−4053.249922	−1515.079764	−4051.899284	−1512.906514	
4	−4111.872771	−1541.588346	−4113.604726	−1542.994444	
5	−3634.242451	−1498.704672	−3632.623418	−1493.600491	
6	−3732.875241	−1501.945109	−3732.359305	−1501.035796	
7	−2679.023828	−1059.868798	−2677.486356	−1058.162716	
8	−462.9435304	41.62243735	−457.1906872	42.73805936	
9	−324.2713339	71.10381988	−323.5365913	71.35703658	
10	−30.41623254	9.09830529	−30.80235656	8.16666604	
Easting difference Correlation Coefficient: 0.9999992; Northing difference Correlation Coefficient: 0.9933340					

## Appendix C. Roadkill Photograph Report

**Table C1:** Animal roadkill recorded by the RRApp categorised by group and species.

Species	Oct.	Nov.	Dec.	Total
**Amphibians**	**4**	**0**	**0**	**4**
eastern sedge frog (*Litoria fallax*)	1	0	0	1
spotted marsh frog, *(Limnodynastes tasmaniensis)*	1	0	0	1
marbled marsh frog (*Limnodynastes convexiusculus*)	1	0	0	1
stony creek frog *(Litoria wilcoxi)*	1	0	0	1
**Diurnal Birds**	**65**	**29**	**16**	**111**
black-faced wood swallow *(Artamus cinereus normani)*	0	2	0	2
black-shouldered kite *(Elanus axillaris)*	0	1	0	1
blue-billed duck *(Oxyura australis)*	0	1	0	1
brush cookoo (*Cacomantis variolosus)*	1	0	0	1
brush turkey *(Alectura lathami)*	11	2	2	15
butcherbird *(Cracticus)*	0	0	1	1
carrawong *(Strepera)*	1	0	0	1
crimson rosella *(Platycercus elegans)*	1	1	0	2
dusky moorhen *(Gallinula tenebrosa)*	1	0	1	2
eastern spinebill (*Acanthorhynchus tenuirostris)*	1	0	0	1
emu *(Dromaius novaehollandiae)*	2	0	0	2
figbird *(Sphecotheres)*	1	0	0	1
forest raven *(Corvus tasmanicus)*	11	2	3	16
galah *(Eolophus roseicapilla)*	5	0	0	5
green rosella *(Platycercus caledonicus)*	0	1	0	1
kookaburra *(Dacelo)*	2	2	4	8
little bronze-cuckoo *(Chrysococcyx minutillus)*	1	0	0	1
little wattlebird *(Anthochaera chrysoptera)*	1	0	0	1
magpie *(Gymnorhina tibicen)*	7	7	0	14
masked lapwing *(Vanellus miles)*	0	2	2	4
noisy friarbird *(Philemon corniculatus)*	1	0	0	1
noisy miner *(Manorina melanocephala)*	4	1	0	5
pied carrawong *(Strepera graculina)*	6	0	0	6
rainbow lorikeet *(Trichoglossus haematodus)*	1	0	0	1
silvereye *(Zosterops lateralis)*	1	0	0	1
spotted pardalote *(Pardalotus punctatus)*	1	0	0	1
sulphur crested cockatoo *(Cacatua galerita)*	1	0	1	2
Tasmanian native hen *(Tribonyx mortierii)*	1	1	0	2
wedge-tailed eagle *(Aquila audax)*	1	1	0	2
whistling kite *(Haliastur sphenurus)*	0	1	0	1
wood duck *(Chenonetta jubata)*	1	2	1	4
white ibis *(Threskiornis molucca)*	1	0	0	1
yellow-tailed black cockatoo *(Calyptorhynchus funereus)*	0	2	1	3
**Exotic Species**	**15**	**5**	**5**	**24**
boar pig *(Sus scrofa domesticus)*	1	0	0	1
cane toad *(Rhinella marina)*	2	1	1	4
cat *(Felis catus)*	2	0	1	3
cow *(Bos Taurus)*	2	0	0	2
deer *(Cervidae)*	0	0	1	1
goat *(Capra hircus)*	0	1	0	1
hare *(Lepus)*	3	2	1	6
rabbit *(Leporidae)*	2	0	1	3
red fox *(Vulpes vulpes)*	1	0	0	1
spotted turtle dove *(Spilopelia chinensis)*	0	1	0	1
call duck *(Anas platyrhynchos)*	1	0	0	1
**Mammal—Macropod**	**412**	**260**	**232**	**904**
agile wallaby *(Macropus agilis)*	19	15	16	50
bandicoot *(Perameles)*	16	2	3	21
Bennet’s wallaby *(Macropus rufogriseus)*	5	6	9	20
bettong *(Bettongia)*	3	0	0	3
eastern grey kangaroo *(Macropus giganteus)*	181	107	89	377
long nose potoroo *(Potorous tridactylus)*	0	0	1	1
Lumholtz’s tree kangaroo *(Dendrolagus lumholtzi)*	1	0	0	1
pademelon *(Thylogale)*	20	17	13	50
parma wallaby *(Macropus parma)*	0	0	1	1
potoroo *(Potorous)*	3	1	0	4
pretty-faced wallaby *(Macropus parryi)*	1	0	0	1
red kangaroo *(Macropus rufus)*	13	9	4	26
red necked wallaby *(Macropus rufogriseus)*	59	30	15	104
red-legged pademelon *(Thylogale stigmatica)*	0	2	1	3
rufous bettong *(Aepyprymnus rufescens)*	1	3	2	6
spectacled hare-wallaby *(Lagorchestes conspicillatus)*	0	1	0	1
swamp wallaby *(Wallabia bicolor)*	51	33	46	130
wallaroo *(Macropus robustus)*	24	22	31	77
western grey kangaroo *(Macropus fuliginosus)*	8	3	1	12
whiptail wallaby *(Macropus parryi)*	7	9	0	16
**Mammal—Other**	**166**	**69**	**46**	**281**
brushtail possum *(Trichosurus vulpecula)*	36	9	4	49
eastern quoll *(Dasyurus viverrinus)*	0	0	2	2
echidna *(Tachyglossidae)*	6	5	2	13
flying fox bat *(Pteropus)*	5	1	2	8
koala *(Phascolarctos cinereus)*	8	4	5	17
ringtail possum *(Pseudocheirus peregrinus)*	4	2	3	9
Tasmanian devil *(Sarcophilus harrisii)*	0	0	1	1
white-tailed rat *(Uromys caudimaculatus)*	0	0	1	1
Wombat *(Vombatidae)*	108	48	25	181
**Nocturnal Birds**	**6**	**3**	**3**	**12**
bush stone-curlew *(Burhinus grallarius)*	0	1	1	2
cassowary *(Casuarius)*	1	0	0	1
eastern barn owl *(Tyto alba)*	2	1	0	3
Papuan frogmouth *(Podargus papuensis)*	0	0	1	1
southern boobook owl *(Ninox novaeseelandiae)*	1	0	0	1
tawny frogmouth *(Podargus strigoides)*	2	1	1	4
**Reptiles**	**52**	**16**	**11**	**79**
bandy bandy snake *(Vermicella annulate)*	2	1	1	4
blotched blue-tongue lizard *(Tiliqua nigrolutea)*	7	4	3	14
bob tail lizard *(Tiliqua rugosa)*	0	2	0	2
broad headed snake *(Hoplocephalus bungaroides)*	0	1	0	1
brown tree snake *(Boiga irregularis)*	1	0	0	1
carpet python (*Morelia spilota)*	4	0	0	4
common tree snake *(Dendrelaphis punctulate)*	1	1	0	2
eastern bearded dragon (*Pogona barbata)*	1	0	0	1
eastern brown snake *(Pseudonaja textilis)*	13	1	2	16
eastern water dragon *(Intellagama lesueurii)*	2	0	1	3
goanna *(Varanus)*	1	0	0	1
keelback snake *(Tropidonophis mairii)*	1	0	0	1
lace monitor lizard *(Varanus varius)*	6	4	1	11
nobbi dragon lizard *(Diporiphora nobbi)*	1	0	0	1
red-bellied black snake *(Pseudechis porphyriacus)*	6	2	1	9
shingleback lizard *(Tiliqua rugosa)*	2	0	0	2
stumped-tailed lizard *(Tiliqua rugosa)*	2	0	0	2
tiger snake *(Notechis scutatus)*	1	0	1	2
western brown snake *(Pseudonaja nuchalis)*	0	0	1	1
yellow spotted monitor *(Varanus panoptes)*	1	0	0	1
